# Bilateral vestibulopathy causes selective deficits in recombining novel routes in real space

**DOI:** 10.1038/s41598-021-82427-6

**Published:** 2021-01-29

**Authors:** Florian Schöberl, Cauchy Pradhan, Maximilian Grosch, Matthias Brendel, Florian Jostes, Katrin Obermaier, Chantal Sowa, Klaus Jahn, Peter Bartenstein, Thomas Brandt, Marianne Dieterich, Andreas Zwergal

**Affiliations:** 1 Department of Neurology, University Hospital, LMU Munich, Munich, Germany; 2 German Center for Vertigo and Balance Disorders, University Hospital, LMU Munich, Marchioninistrasse 15, 81377 Munich, Germany; 3 Department of Nuclear Medicine, University Hospital, LMU Munich, Munich, Germany; 4 Neurological Hospital, Schön Klinik Bad Aibling, Bad Aibling, Germany; 5 Clinical Neurosciences, University Hospital, LMU Munich, Munich, Germany; 6 Munich Cluster of Systems Neurology, SyNergy, Munich, Germany

**Keywords:** Neuroscience, Physiology, Diseases, Neurology

## Abstract

The differential impact of complete and incomplete bilateral vestibulopathy (BVP) on spatial orientation, visual exploration, and navigation-induced brain network activations is still under debate. In this study, 14 BVP patients (6 complete, 8 incomplete) and 14 age-matched healthy controls performed a navigation task requiring them to retrace familiar routes and recombine novel routes to find five items in real space. [^18^F]-fluorodeoxyglucose-PET was used to determine navigation-induced brain activations. Participants wore a gaze-controlled, head-fixed camera that recorded their visual exploration behaviour. Patients performed worse, when recombining novel routes (p < 0.001), whereas retracing of familiar routes was normal (p = 0.82). These deficits correlated with the severity of BVP. Patients exhibited higher gait fluctuations, spent less time at crossroads, and used a possible shortcut less often (p < 0.05). The right hippocampus and entorhinal cortex were less active and the bilateral parahippocampal place area more active during navigation in patients. Complete BVP showed reduced activations in the pontine brainstem, anterior thalamus, posterior insular, and retrosplenial cortex compared to incomplete BVP. The navigation-induced brain activation pattern in BVP is compatible with deficits in creating a mental representation of a novel environment. Residual vestibular function allows recruitment of brain areas involved in head direction signalling to support navigation.

## Introduction

Target-oriented navigation relies on visual information from the environment (allothetic cues), taking into account simultaneous vestibular and somatosensory afferent inputs (idiothetic cues) for continuous updating of one’s own position in space during locomotion^1^. Multisensory inputs are equally relevant for ego- and allocentric navigation strategies^2,3^. Egocentric navigation is based on a compass-like strategy, where the navigator’s current position in space is the absolute reference point for all the objects in the surrounding environment^2,4,5^. This strategy is often used to retrace familiar routes. In contrast, allocentric navigation relies on a map-like strategy, where different objects of the environment can be set in relation to each other independent of the navigator’s current position in space^6,7^. This strategy is most appropriate for recombining novel routes. During real-space navigation there is continuous overlap and change of ego- and allocentric strategies^3^. Navigation is guided by a widespread network of brain regions (prefrontal cortex, basal ganglia, thalamus, cerebellar regions, posterior parietal cortex, retrosplenial cortex, posterior parahippocampus, lingual gyrus, hippocampus, and entorhinal cortex)^7,8^. Creating a mental representation of a novel environment seems to depend critically on the hippocampus, entorhinal cortex, and retrosplenial cortex with its highly specialized cell ensembles (place cells, grid cells, and head direction cells)^3,7,9^.

Bilateral vestibular damage in rodents leads to severe and persistent navigation deficits in real space by disrupting the head direction cell code in the dorsal brainstem tegmentum, anterior thalamus, subiculum and entorhinal cortex, the place cell code in the hippocampus, and the grid cell code in the entorhinal cortex^10–13^. Patients with complete bilateral vestibulopathy (BVP) show spatial memory deficits in a desktop-based virtual variant of the Morris Water Maze Task (vMWMT) accompanied by a significant hippocampal volume loss^14^. Delayed spatial learning performance in vMWMT and a decrease in gray matter hippocampal/parahippocampal volume were also found in patients with incomplete BVP^15^. However, in a virtual cityscape navigation paradigm, patients with incomplete BVP had no significant performance deficits, and exhibited increased activations in the posterior cerebellum^16^. The authors interpreted these findings as a change of the prevailing navigation strategy towards sequence-wise learning of certain routes by loops in the cerebellum, basal ganglia, and prefrontal cortex^17^.

**Figure 1:**
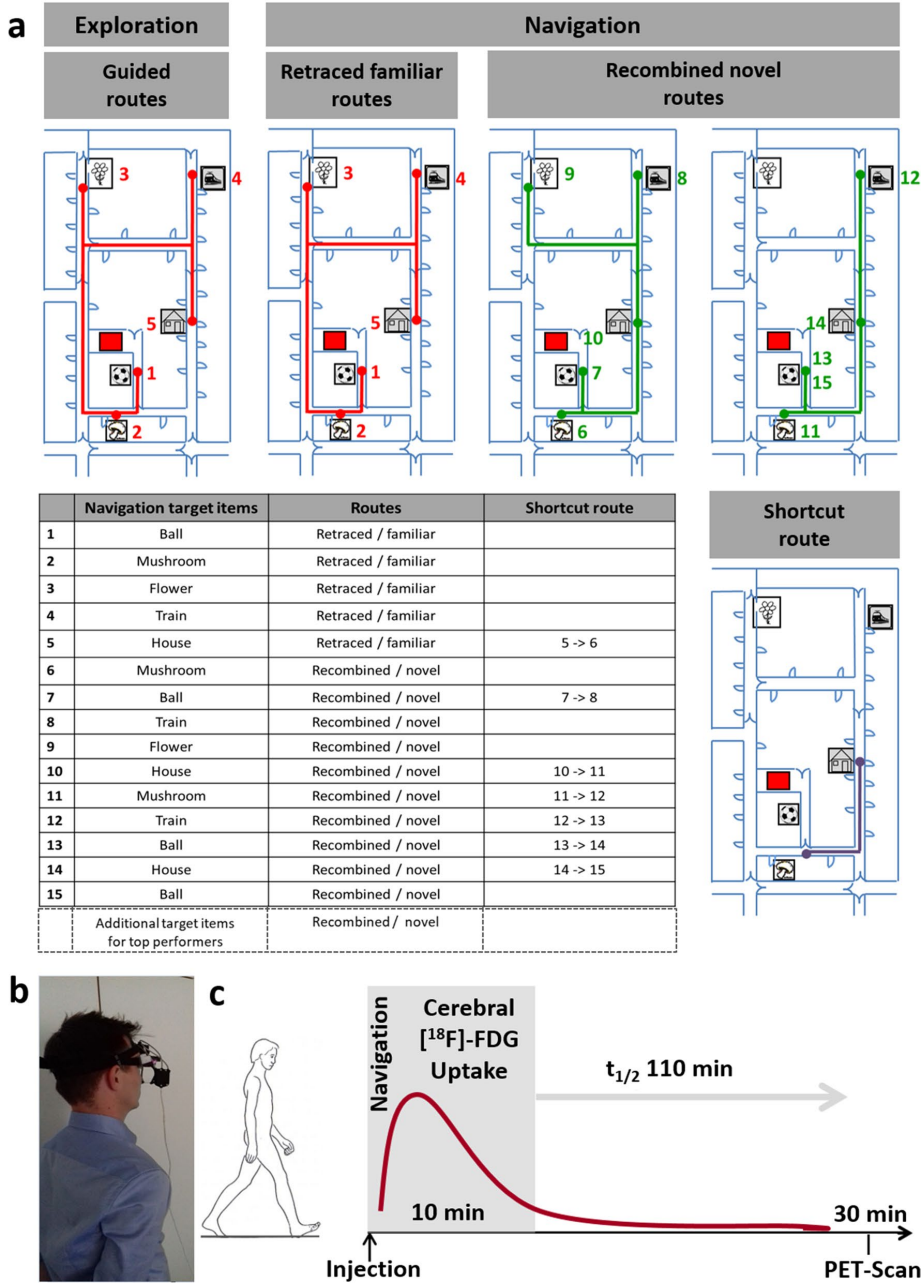
Navigation paradigm in real space. (**a**) All subjects performed a navigation paradigm in a complex and unfamiliar spatial environment to test their spatial orientation performance. The area, in which five target items (pictures of ball/mushroom/flower/train/house) had been placed, was shown to the subjects first on an investigator-guided walk (exploration, upper panel, left side). Afterwards subjects had to find the items in a defined pseudo-randomised order over the next 10 min beginning from the starting point (red square). The first five routes in the navigation paradigm were identical to the previous exploration routes and therefore had to be simply retraced (referred to as retraced familiar routes in the manuscript) (upper panel, middle). Then, the order of target items was changed in a way that required recombining novel routes (referred to as recombined novel routes in the manuscript) (upper panel, right side). Potential shortcut routes were registered (lower panel, right side). The sequence of items during navigation is depicted in a table (lower panel, left side) and appears as numbers beside the target items in the figures. (**b**) Subjects wore a gaze-monitoring head camera throughout the experiment to allow post-hoc analysis of their visual exploration. (**c**) [^18^F]-FDG was injected at the start of the 10-min navigation phase. After the end of navigation testing subjects rested in a supine position for 20 min and image acquisition started 30 min after tracer administration. By this method navigation-induced brain activations could be depicted because the cerebral glucose utilisation is weighted to the 10 min following [^18^F]-FDG injection and is integrative due to intracellular trapping of the tracer (adapted from reference ^19^^,^^21^).

Desktop-based virtual reality (VR) setups may not be the optimal choice to study the vestibular contribution to spatial navigation, because they only test the 2-dimensional static vestibular spatial memory and are predominantly visually guided^18,19^. In comparison, real-space navigation allows for multisensory and specifically 3D vestibular inputs induced by translational and rotational head and body movements^1,20^. Therefore, the aim of the current study was to investigate the role of bilateral vestibular deafferentation (either complete or incomplete) on navigation behaviour and brain activations under a real-world multisensory setting. By simultaneous eye-tracking and [^18^F]-fluorodeoxyglucose (FDG)-PET imaging during a real-space navigation task^21^, we analyzed navigation performance, visual explorations, travelled paths, and navigation-induced brain activations in a group of 14 BVP patients (complete n = 6; incomplete n = 8) in comparison to 14 age-matched healthy controls. The real-space navigation task consisted of an investigator-guided exploration phase (10 min) directly followed by a navigation phase (10 min), in which participants had either to retrace familiar routes, or recombine novel routes (Fig. 1). Based on previous findings in rodents and humans, we hypothesized that BVP patients show more deficits in recombining novel routes than controls due to reduced navigation-induced hippocampal activation, and that these alterations critically depend on the extent of vestibular dysfunction.

## Results

### Patient characteristics

BVP patients (n = 14, 54.1 ± 12.2 years, 7 female) were classified as complete BVP (cBVP, n = 6, horizontal vHIT gain: 0, caloric response: 0 deg/s, missing o/cVEMP response) or incomplete BVP (iBVP, n = 8, horizontal vHIT gain: 0.44 ± 0.13, caloric response: 2.2 ± 1.18 deg/s, oVEMP pathological in n = 5, cVEMP pathological in n = 6), and compared to age-matched healthy controls (HC) (n = 14, 55.1 ± 10.1, 7 female). Patients with cBVP were younger (46.5 ± 7.4 years) than those with iBVP (63.3 ± 10.5 years) (p = 0.02). The duration of bilateral vestibular failure was comparable between both groups (cBVP: 8.0 ± 2.6 years; iBVP: 8.3 ± 2.4 years; p = 0.78). The etiology of cBVP was bilateral vestibular neurectomy for treatment of vestibular schwannomas due to neurofibromatosis type 2 in all cases, while iBVP was classified as toxic (gentamicin) (n = 3) or idiopathic (n = 5) (Supplementary Table S1). Educational level did not differ between groups (BVP: 12.4 ± 2.2 years; HC: 12.5 ± 2.4 years; p = 0.93). Cognitive deficits were excluded by Montreal Cognitive Assessment (MOCA) (BVP: 29.3 ± 0.8; HC: 29.2 ± 0.8; p = 0.84).

### Navigation performance of BVP patients and healthy controls

**Figure 2:**
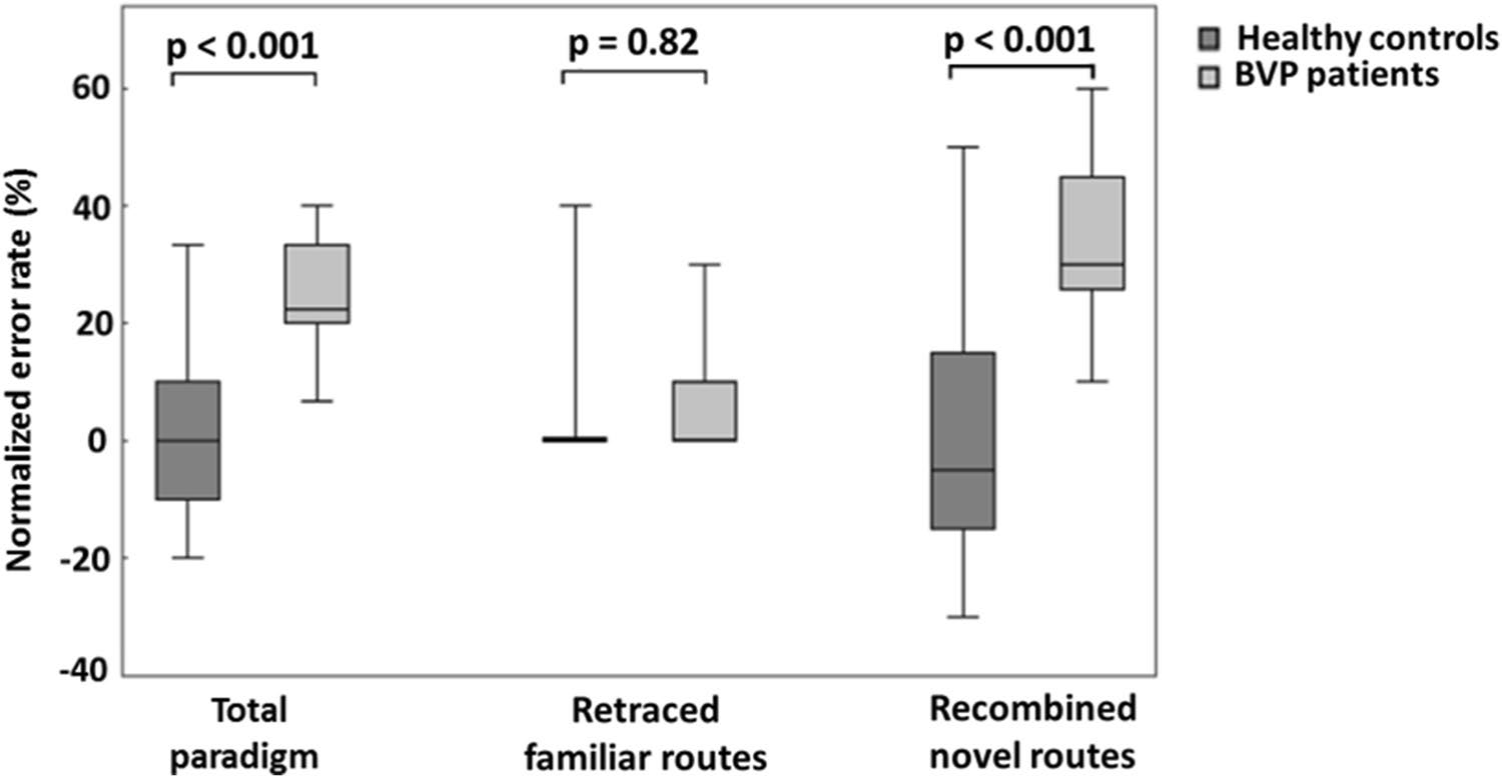
Navigation performance in BVP patients and healthy controls. The total normalized error rate during the navigation paradigm was significantly higher in BVP patients compared to healthy controls. BVP patients made more errors when recombining novel routes only, while they performed equally on retracing familiar routes. Normalized error rates are depicted as Tukey plots in %. BVP: bilateral vestibulopathy.

Normalized error rates during real-space navigation (see “Methods”) were higher in BVP patients (29.0 ± 11.2%) than in controls (2.8 ± 5.3%) (p < 0.001). This was due to BVP patients making more errors when recombining novel routes (37.5 ± 16.1% versus 1.7 ± 5.8%) (t = 7.3, p < 0.001). On retraced, i.e., familiar routes, BVP patients and controls performed equally well (5.0 ± 12.4% versus 6.2 ± 11.9%) (t = 0.2, p = 0.82) (Fig. 2). Nevertheless, the navigation performance of the BVP patients was far better than random search (Supplementary Table S2). Gait velocity during steady-state locomotion was the same for BVP patients and controls (1.2 ± 0.1 m/s vs. 1.3 ± 0.1 m/s) (t = 0.4, p = 0.69). Correlation analysis revealed that the normalized error rate for recombined novel routes significantly increased with lower mean caloric response (Rho  = − 0.66, p = 0.03) and lower mean horizontal vHIT gain (Rho = − 0.91, p = 0.01) in iBVP patients (Supplementary Fig. 1). The normalized error rate for all routes was not significantly correlated with caloric response or horizontal vHIT gain.

### Exploration behaviour and navigation strategy in BVP patients and healthy controls

**Figure 3:**
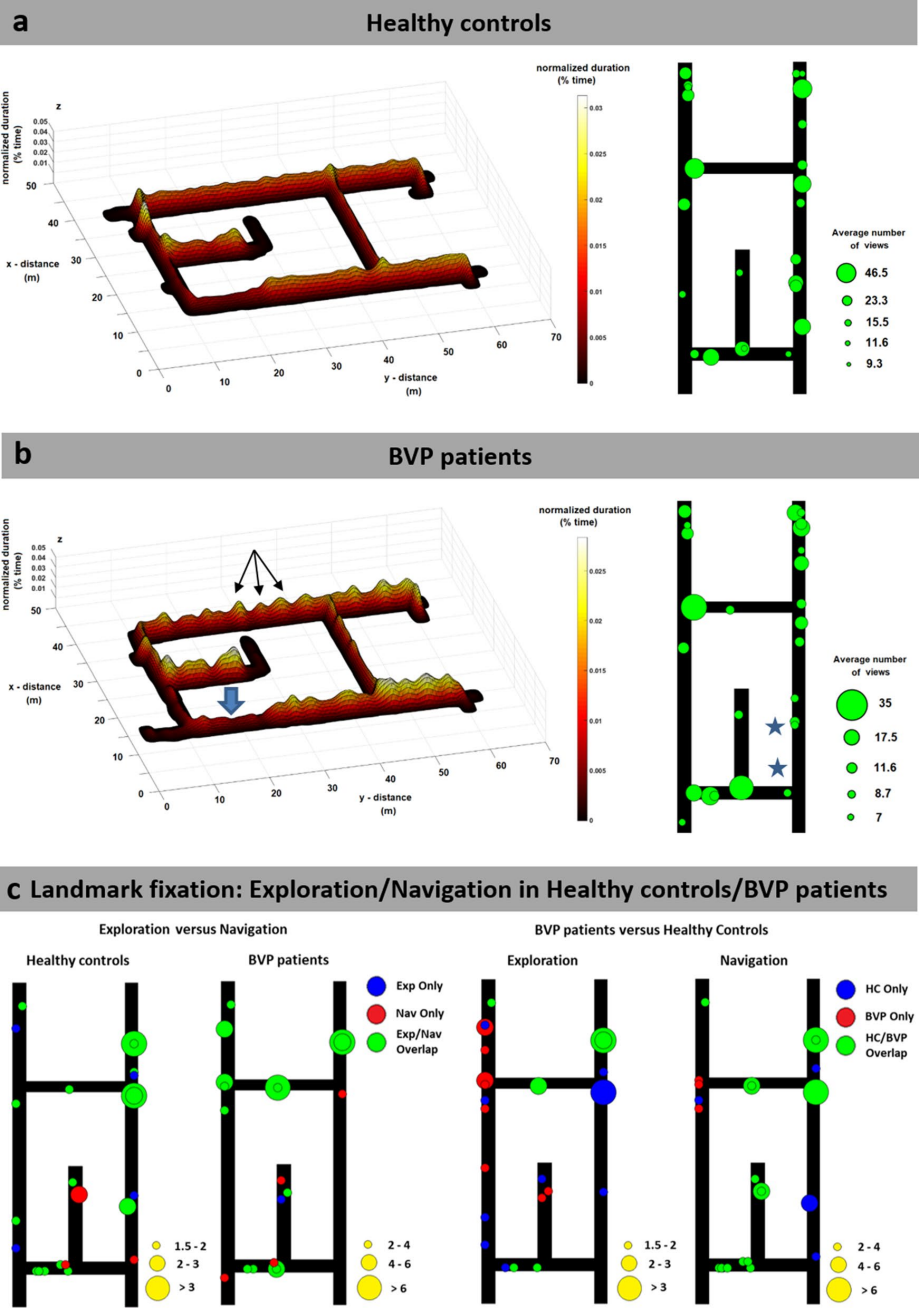
Navigation strategy and visual exploration patterns in healthy controls and BVP patients. (**a**) Controls showed a navigation strategy that included the use of shortcuts, indicating the presence of a mental representation of the environment with a metric scale (i.e., cognitive map). (**b**) In contrast, BVP patients used shortcuts less frequently for path optimisation (blue arrow), and instead spent more time on familiar routes. Their movement along the path was highly characteristic and dominated by fluctuations (stop-and-go pattern, black arrows). BVP exhibited fewer visual fixations along the possible shortcut route (blue stars). The search path during navigation was colour coded on a ground map of the navigation paradigm in real space (x, y) as normalized duration (% of time). The position of the 25 most frequently fixated objects feasible as landmarks in each group was indicated on a ground map as green circles (diameters representing the average number of views to a respective object across the whole group). (**c**) The overlap of fixations to specific landmarks was compared quantitatively within groups (for exploration versus navigation) (left side) and within the exploration and navigation paradigms (for HC versus BVP group) (right side). The green circles indicate an overlap of fixated objects in both conditions (left: exploration and navigation; right: HC and BVP group), the blue and red circles objects fixated only during one condition (left: blue—exploration only, red—navigation only; right: blue—HC only, red—BVP only). Diameters of circles represent the average number of object fixations (scales illustrated as yellow circles, respectively). BVP: bilateral vestibulopathy, HC: healthy controls.

**Figure 4:**
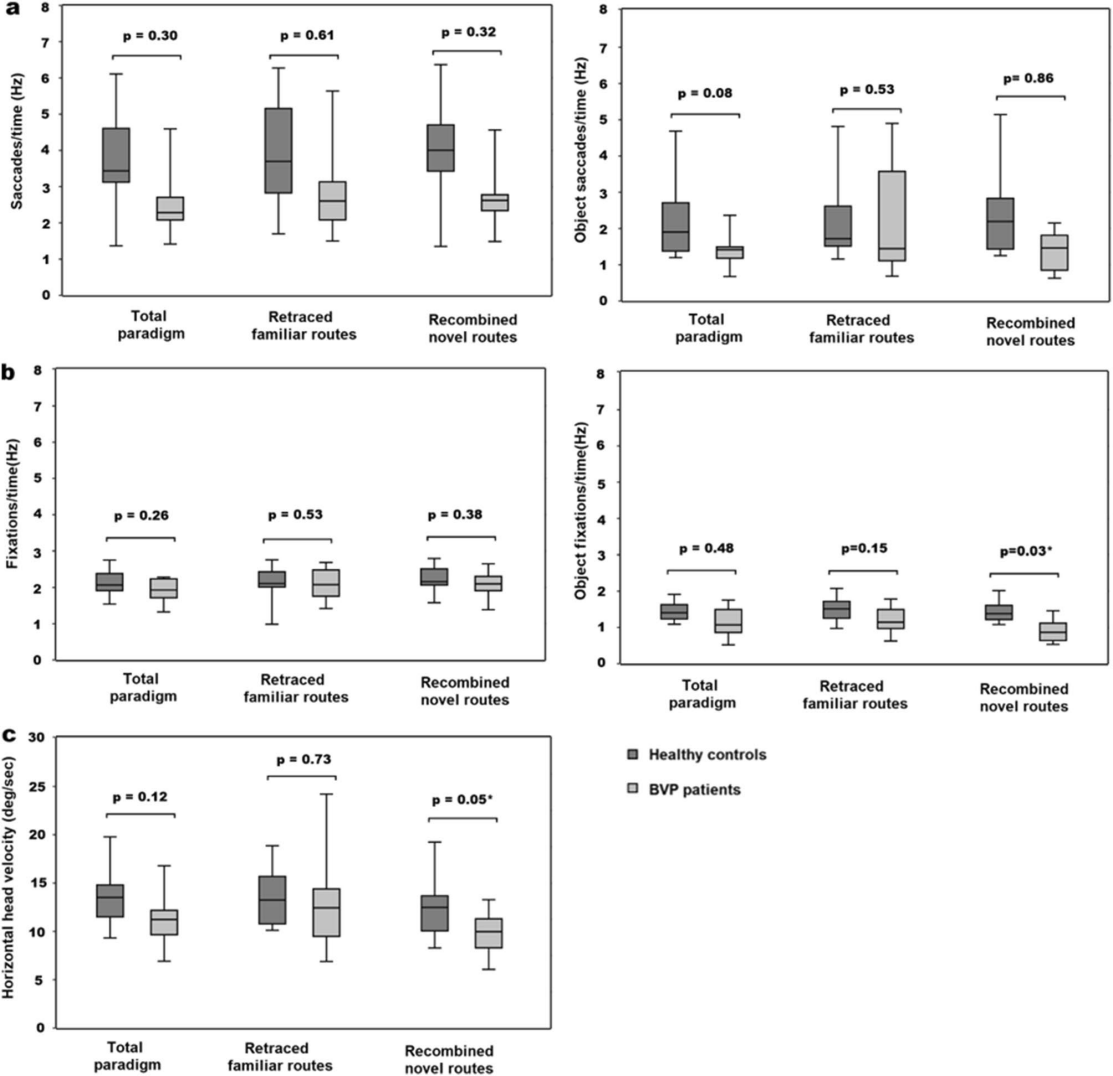
Eye and head movement parameters in healthy controls and patients with BVP. (**a**) Number of saccades/time (Hz) (total, retraced familiar routes, recombined novel routes) did not differ between BVP and HC. Number of saccades/time (Hz) (total, retraced familiar routes, recombined novel routes) towards objects feasible as landmarks also did not differ between BVP and HC. (**b**) Number of fixations/time (Hz) (total, retraced familiar routes, recombined novel routes) did not differ between BVP and HC. Number of object-specific fixations/time (Hz) when recombining novel routes was significantly lower in the BVP, while object-specific fixations in total and on retraced familiar routes were comparable between BVP and HC. (**c**) Horizontal head velocity (deg/s) was lower in BVP, when recombining novel routes, and similar when retracing familiar between both groups. The values are depicted as Tukey plots, respectively. BVP: bilateral vestibulopathy, deg: degree, HC: healthy controls, Hz: Hertz.

The search path was profoundly different between both groups: BVP patients spent significantly less time at crossroads (t = 4.9, p < 0.001) during the whole navigation paradigm (Table 1). Furthermore, they used the possible shortcut route less often compared to controls (Table 1; Fig. 3a,b). The movement along the path showed high fluctuations in BVP with a characteristic “stop-and-go like locomotion pattern” (Fig. 3b). When calculating the travelled distances in relation to the optimal (i.e., shortest) path, there was no significant difference between BVP and HC (t = 0.80, p = 0.53) (Table 1). The visual exploration behaviour in BVP patients also differed from that of HC. For the sake of a comprehensive analysis, we differentiated fixations to objects suitable as spatial landmarks from non-specific fixations (e.g., to bare wall, floor), task-specific fixations (e.g., to the investigator), and fixations reflecting non-specific search behaviour (e.g., to doors) (see “Methods”). When object fixations to specific landmarks were compared during the exploration and navigation phase, HC had an overlap of 68% and BVP patients of 80%, indicating a similar individual recall of objects relevant for spatial orientation in both groups (p = 0.52) (Fig. 3c, left). The overlap of distinct object fixations between groups was 68% during navigation. HC had more object fixations along the shortcut route, BVP patients at crossroads (Fig. 3c, right). In the next step, we analyzed the number of fixations and saccades quantitatively for the total paradigm and separately for retraced familiar routes (known from exploration), and recombined novel routes (not known from exploration). To correct for possible differences in locomotion speed on the single routes, fixations and saccades were expressed in Hz (absolute values/s). Saccades, fixations, and total horizontal head movements during the total navigation task were comparable between BVP patients and HC (Table 1, Fig. 4). During retracing of familiar routes, overall saccades (t = 0.6, p = 0.61), object-specific saccades (t = 0.18, p = 0.53), fixations (t = 0.18, p = 0.53), object-specific fixations (t = 1.5, p = 0.15), and horizontal head movement velocity (t = 0.4, p = 0.73) were similar in both groups (Table 1). However, BVP patients exhibited significantly fewer object fixations (t = 3.6, p = 0.03) and lower horizontal head movement velocity (t = 2.0, p = 0.05) than HC when recombining novel routes (Table 1; Fig. 4b,c). No statistically relevant differences appeared between both groups for saccades, fixations, and horizontal head movement velocity, if only standing or walking phases were analyzed (Supplementary Table S3). Horizontal head movement velocity was not different between retraced familiar and novel recombined routes within the group of BVP patients (t  = − 1.7, p = 0.12) and the group of HC (t = 1.7, p = 0.13).

**Table 1:** Visual exploration and navigational parameters in healthy controls and BVP patients.

Parameter	HC	BVP	Independent t-test (t , p) with Bonferroni-correction
Duration at crossroads (%)	21.1 ± 2.6	14.1 ± 3.7	4.9, < 0.001*
Use of shortcut route (%)	58.2 ± 18.4	28.9 ± 18.9	14.0, 0.01*
Travelled distance/optimal path (%)	108.6 ± 9.1	114.6 ± 18.5	0.80, 0.53
Total paradigm: saccades/time (Hz)	4.2 ± 1.8	2.5 ± 0.9	2.4, 0.30
Total paradigm: fixations/time (Hz)	2.1 ± 0.33	1.9 ± 0.35	1.3, 0.26
Total paradigm: object saccades/time (Hz)	2.3 ± 1.2	1.4 ± 0.51	1.9, 0.08
Total paradigm: object fixations/time (Hz)	1.4 ± 0.26	1.1 ± 0.44	1.9, 0.48
Familiar routes: object saccades/time (Hz)	2.3 ± 1.1	2.2 ± 1.7	0.18, 0.53
Familiar routes: object fixations/time (Hz)	1.5 ± 0.34	1.2 ± 0.43	1.5, 0.15
Novel routes: object saccades/time (Hz)	2.5 ± 1.1	2.2 ± 1.7	0.18, 0.86
Novel routes: object fixations/time (Hz)	1.5 ± 0.28	0.92 ± 0.33	3.6, 0.03*
Total paradigm: horizontal head velocity (deg/s)	13.4 ± 2.9	11.2 ± 3.0	1.6, 0.12
Familiar routes: horizontal head velocity (deg/s)	13.6 ± 2.8	13.0 ± 5.2	0.4, 0.73
Novel routes: horizontal head velocity (deg/s)	12.3 ± 3.1	9.8 ± 1.9	2.0, 0.05*

The duration at crossroads and use of the possible shortcut routes were significantly lower in the BVP group. Total saccades and fixations, as well as fixations and saccades to objects feasible as landmarks for orientation did not differ between both groups. When retracing familiar routes (routes shown during the exploration phase), object fixations and saccades were similar between both groups. In contrast, when recombining novel routes (routes not shown during the exploration phase) object fixations were lower in the BVP group. Total horizontal head movements and horizontal head movements on familiar routes were comparable between both groups. However, there were fewer horizontal head movements in the BVP group on recombined novel routes.

BVP: bilateral vestibulopathy, deg: degree, HC: healthy controls, Hz: Hertz.

*Significant inter-group differences after applying Bonferroni-correction for multiple testing.

### Cerebral glucose metabolism during navigation in BVP patients and healthy controls

**Figure 5:**
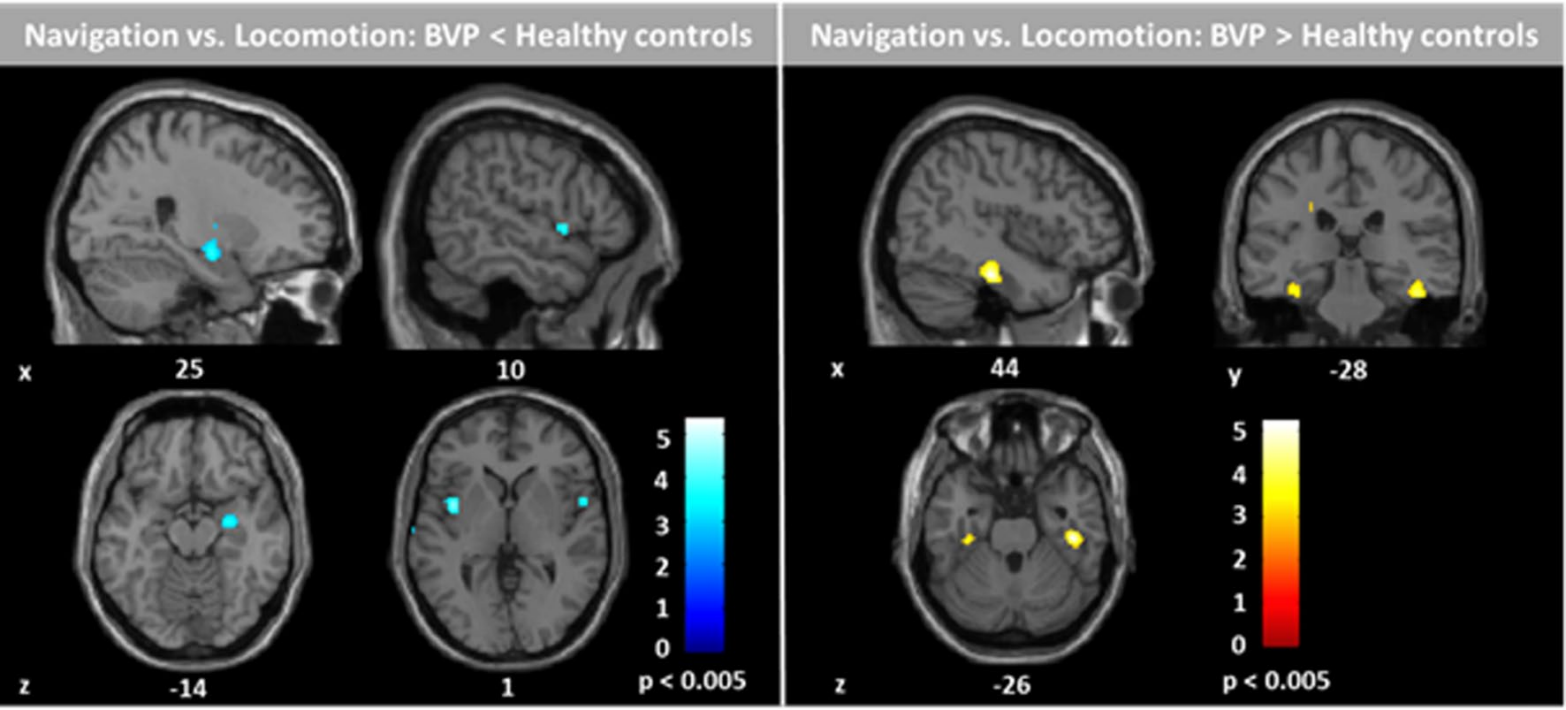
Regional cerebral glucose metabolism (rCGM) during navigation compared to locomotion in healthy controls and BVP patients. A direct comparison between groups for the navigation versus locomotion condition revealed a significant decrease of rCGM in the right hippocampal formation and bilateral insular cortex, and a significant increase of rCGM in the bilateral parahippocampus and lingual gyrus (i.e., parahippocampal place are, PPA) in BVP patients compared to healthy controls. Significance level: p < 0.005; colour scale indicates t-score; levels of sections in x- and z-direction are given by MNI coordinates. BVP: bilateral vestibulopathy.

**Figure 6:**
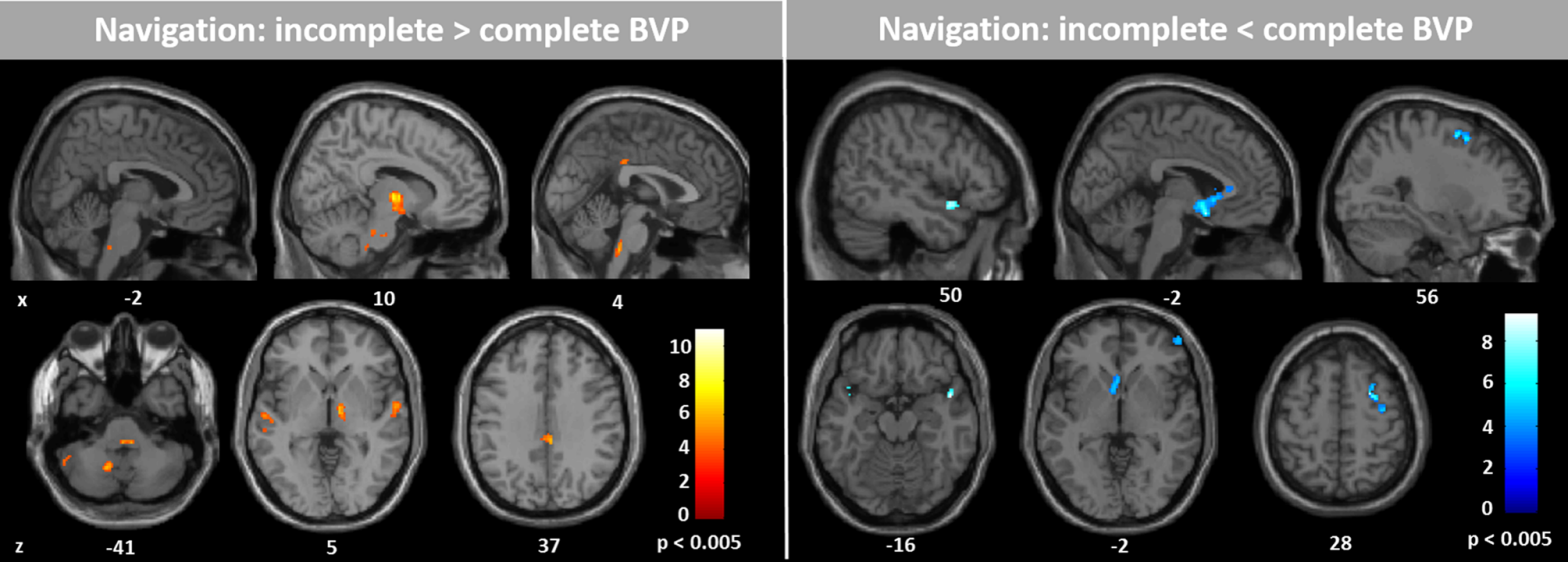
Regional cerebral glucose metabolism (rCGM) during navigation in BVP subgroups. Comparison of rCGM during navigation in patients with complete (mean vHIT gain: 0, mean caloric response: 0 deg/s) and incomplete BVP (mean vHIT gain: 0.4, mean caloric response: 2.2 deg/s) revealed the following: patients with incomplete BVP had higher rCGM in the pontine brainstem tegmentum, vestibulocerebellum (left > right flocculus), anterior thalamus (right > left), and retrosplenial cortex, and lower rCGM in the dorsolateral prefrontal cortex, subgenual prefrontal cortex, superior temporal gyrus, and caudate nucleus. Significance level p < 0.005; colour scale indicates t-score; levels of sections in x- and z-direction are given by MNI coordinates. BVP: bilateral vestibulopathy.

A direct comparison of rCGM during navigation versus locomotion of HC and BVP patients revealed a relative decrease of rCGM in the right anterior hippocampus and bilateral insular cortex, and a relative increase of rCGM in the posterior parahippocampal cortex and lingual gyrus (i.e., parahippocampal place area, PPA) bilaterally in BVP patients (Fig. 5). Directly comparing the two subgroups cBVP versus iBVP revealed a significantly higher rCGM in the pontine brainstem tegmentum, vestibulocerebellum, anterior thalamus, posterior insular, and retrosplenial cortex in iBVP patients. In contrast, iBVP exhibited lower rCGM in the superior and medial frontal gyrus, the subgenual prefrontal cortex, superior temporal gyrus, and caudate nucleus as compared to cBVP (Fig. 6). Similar results were found, when correlating residual vestibular function by vHIT gain with rCGM across the entire BVP group (Supplementary Fig. S2). Correlation of the percentage use of shortcut with rCGM revealed an increased activation of the pontine brainstem tegmentum, vestibulocerebellum, and right anterior thalamus, as well as a decreased activation of the prefrontal cortex areas with more frequent use of the shortcut route (Supplementary Fig. S3).

## Discussion

The major findings for navigation performance, visual exploration behaviour, search path, and brain activations in patients with BVP were as follows: (1) BVP resulted in a selective impairment of recombining novel routes in a real-space environment, which correlated with the degree of vestibular hypofunction. (2) BVP patients exhibited higher gait fluctuations with a “stop-and-go like locomotion pattern”, spent less time at crossroads and used a possible shortcut route less frequently. (3) Patients showed significantly fewer object fixations and horizontal head movements, when recombining novel routes. (4) The described alterations in navigation performance, search path, visual fixations and horizontal head movements were accompanied by reduced navigation-induced activations in mesiotemporal brain regions such as the hippocampus and entorhinal cortex; in contrast, there were higher activations in posterior mesiotemporal and temporooccipital (posterior parahippocampus and lingual gyrus) brain regions. (5) Navigation-induced brain activations significantly differed in patients with cBVP compared to those with residual vestibular function. iBVP patients exhibited higher activations in the pontine brainstem tegmentum, anterior thalamus, retrosplenial and posterior insular cortex, while cBVP patients showed higher activations in the caudate nucleus, dorsolateral prefrontal cortex and subgenual prefrontal cortex.

### Real-space navigation performance and behaviour in BVP patients

BVP patients performed comparably to HC, when they had to retrace familiar routes, which had been shown before during an examiner-guided walk through the novel environment. However, they exhibited significant deficits, when recombining novel routes within this environment. Nevertheless, the navigation performance in BVP patients was clearly better than expected from a random-search strategy. Overall, the reduced navigation performance of BVP patients for novel route finding in our real-space task is in line with results from previous studies using desktop-based VR setups^14,15^. Only one recent study did not find differences for shortest route finding in a virtual cityscape in patients with severe, but incomplete BVP as compared to healthy age-matched controls^16^. A reasonable explanation for these seemingly controversial results might be the design and nature of the applied navigation tasks. The virtual cityscape paradigm of Jandl et al.^16^ induced a strong bias towards allothetic visual cues^9,22^ to find the most direct routes to target items, while processing and neural integration of idiothetic cues (i.e., vestibular, somatosensory inputs, motor-efference copy signals) was less relevant. The latter play an important role in continuous updating of the current position in space by path integration^23–25^. Furthermore, the route-learning task in their virtual environment could be absolved correctly without a mental representation or cognitive map of the cityscape. Abundant landmarks at most crossroads on the requested routes led to a bias towards stimulus–response like route learning (i.e., coupling of certain landmarks with a direction decision)^26–29^. Regarding all these details, the virtual navigation task by Jandl et al.^16^ is quite similar to the first part of our real-space navigation task, where familiar routes had to be retraced (Fig. 1a). Congruent to their results, navigation performance under these preconditions was similar between BVP patients and HC (Fig. 2). Other previous studies used a virtual version of the Morris Water Maze Task (vMWMT) to study navigation in BVP, which is less enriched by visual cues and thus less dependent on landmark processing^14,15^. Furthermore, vMWMT is suitable to examine also non-egocentric or non-stimulus-responses like spatial learning and memory by a variable change of starting position and a need for recombining novel trajectories to get to the requested hidden platform^30,31^. Spatial precision thus is of great relevance for the vMWMT^32^. One major strategy to solve the vMWMT is the recombination of novel trajectories to reach the target items, similar to the second part of our real-space navigation paradigm (Fig. 1a). In this aspect, BVP patients consistently performed worse than HC (Fig. 2). However, we recognize that vMWMT can be solved in multiple ways also without recreating novel trajectories.

In the current real-space navigation task, we cannot completely exclude an effect of the order of the routes on navigation performance, which means that BVP patients perform worse than HC on the later routes for example due to faster and more prominent tiredness. However, this seems rather unlikely, since BVP patients had no problems with their physical condition during stereotyped hallway locomotion for 20 min on a second date. Furthermore, none of the BVP patients showed signs of or reported relevant tiring or fatigue during or after the navigation task. We also can definitely exclude (verbal) memory deficits as a confounder for the observed navigation deficits in BVP patients, as each patient could recall the five target items after the exploration and navigation phase without problems. In line with this, MOCA screening revealed normal cognitive abilities in each subject within both groups (BVP and HC).

We favour the view that the selective deficits of BVP patients in recombining novel routes are a direct correlate of an altered navigation behaviour due to deficient vestibular input and processing. BVP patients spent significantly less time at four of the five crossroads within the real-space environment. Furthermore, they exhibited fewer fixations towards objects feasible as landmarks for (re)orientation and performed fewer horizontal head movements exclusively when recombining novel routes. They used the strategic shortcut route significantly less frequently than the HC, which is a major clue for different navigation behaviours between both groups. The gait patterns between BVP patients and HC differed profoundly. BVP patients exhibited a characteristic “stop-and-go like gait pattern” during the navigation task. One might claim that all these changes are just a consequences of a deficient vestibulo-ocular reflex (VOR) in BVP, which may lead to reduced eye and head movements and more instability under dynamic walking conditions^33^. However, we do not think that a deficient VOR alone can account for all these behavioural differences during real-space navigation. Overall saccades, fixations, and horizontal head movements on retracing familiar routes did not differ between BVP patients and HC. Finally, there was no difference in saccades per time towards objects feasible as landmarks, when recombining novel routes, which would be expected, if a deficient VOR were the only explanation. Taking into account the reduced duration at crossroads and the use of shortcuts, it seems more likely, that these changes indeed reflect a distinct navigation behaviour of BVP patients. In recognition of previous studies and theoretical concepts on human navigation, the navigation-specific behaviour in BVP patients might be explained in three ways: (1) Impaired topological knowledge, i.e. heuristics, which essentially means difficulties in choosing the most suitable navigation strategy for a specific situation or task independent of Cartesian-like metric representations^3^. (2) A deficit in creating a mental representation or cognitive map of the novel environment^7^. (3) A noisier and less accurate path integration due to incorrect simultaneous processing of multiple idiothetic (i.e., vestibular, somatosensory inputs and motor-efference copy) cues. Mental representation and path integration are essential abilities to recombine novel routes and particularly find the most optimal route to a target item in a novel environment^1,20,34^. The characteristics of the chosen environment (largely visible environmental space, rectangular geometry, restriction by environmental borders) and the design of our task (a clear separation into two parts with retracing familiar routes and recombining novel routes) are not optimal to examine the role of topological knowledge^3^. However, they are appropriate to differentiate stimulus–response like route processing from mental capturing of the spatial layout and path integration by simultaneous processing of allo- and idiothetic cues^34,35^. The correlation of a higher residual vestibular function in the high- and low-frequency spectrum with a better ability to recombine novel routes in the BVP group further implies that the observed differences between groups are more than just the direct consequence of a VOR deficit. Whether this correlation rather favours a deficit in precise path integration or mental representation of the environment as a cause of navigation deficits in BVP cannot be disentangled finally by our study.

### Navigation-induced brain activation patterns in BVP patients

A complex and widely distributed cerebral network guides specific navigation strategies in humans: the parahippocampal place area (PPA) for landmark processing^9,22,36^; the posterior parietal cortex (PPC), (pre)frontal cortex, striatum, and cerebellum for distance estimation^17,29^; the retrosplenial cortex (RSC), precuneus, and anterior dorsal nucleus (ADN) of the thalamus for direction computations via the head direction cell code^37–39^; and the anterior hippocampus, and entorhinal cortex for the creation of a metric cognitive map of the environment by specific cell types, such as place and grid cells^40–45^.

In the current study, BVP patients had a reduced activation in the right anterior hippocampus and bilateral insular cortex, and a higher activation in the posterior parahippocampus and lingual gyrus (PPA) than controls, when comparing navigation against stereotyped hallway locomotion (Fig. 5). Previous studies in rodents and humans showed that vestibular afferents project to the anterior hippocampus via multiple pathways^20,46–49^. Thus, less vestibular input due to BVP could lead to reduced hippocampal activation irrespective of navigation. However, in a previous study, suprathreshold galvanic vestibular stimulation evoked brain activations in the insular cortex, superior temporal gyrus and inferior parietal areas, but not in the hippocampus^50^. This study supports the view that vestibular signals during rotational or translational head movements will not lead to hippocampal activations per se. Instead, the multiple vestibulo-hippocampal and vestibulo-entorhinal connections seem to become important only for higher cognitive vestibular functions such as path integration and navigation^20,46,51^. Plausible cellular correlates are the hippocampal place cells, which encode specific locations in an environment via place fields, and the grid cells and head direction cells in the entorhinal cortex, which encode distances and directions between different locations of surrounding space^40,52,53^. These cell types receive multimodal sensory, particularly visual and vestibular inputs^20,54,55^. Disintegrated place cell ensembles or a disruption of the hippocampal theta rhythm due to absent vestibular input could explain hippocampal dysfunction during spatial orientation in BVP. Therefore, it seems likely that a reduced navigation-induced activation of the right hippocampus in BVP patients is a direct functional correlate of an impaired mental representation of the novel environment or a deficient, since too noisy path integration^7,34,56^. Accordingly, a recent study showed that hippocampus-dependent navigation improved after enhancing vestibular afferent inputs by galvanic vestibular stimulation in healthy subjects, while stimulus–response like spatial learning remained unaffected^57^. The direct correlation of decreased hippocampal activation with a higher error rate on recombined novel routes in our study further strengthens that view. Interestingly, navigation-induced hippocampal activation did not directly correlate with quantitative parameters of vestibular hypofunction (vHIT gain, caloric response) in the current study. It is likely that vestibular inputs to place cells are already integrated from angular velocity signals, which would arise from semicircular canals (SCC) in the different planes, to space direction-specific signals, which are computed by various sources of vestibular signals^58^.

Increased navigation-induced activation of the bilateral PPA in BVP patients might reflect a compensatory mechanism. In consequence of deficient hippocampal place learning, increased visual scene processing and visual analyses of the novel environment seem to be a reasonable strategy to overcome the aforementioned deficits. One might argue that fewer fixations towards objects feasible as landmarks during recombining novel routes in the BVP group contradict this view. However, previous studies showed that the PPA is critically involved in processing novel visual scenes and the selection of visual cues as landmarks^59,60^. A higher activation of PPA thus would be compatible with a more appropriate selection instead of a higher quantity of landmarks. The incorporation and optimal employment of selected landmarks for precise navigation is not driven by the PPA, but instead by the RSC^9,22^.

Comparison of cBVP and iBVP subgroups revealed further significant differences in navigation-induced brain activation patterns. iBVP patients exhibited a higher rCGM in the pontine brainstem tegmentum, vestibulocerebellum, anterior thalamus and retrosplenial cortex, whereas cBVP patients had higher activations in the caudate nucleus, dorsolateral prefrontal cortex and subgenual prefrontal cortex (Fig. 6). When correcting for the brain activations during stereotyped locomotion, these differences persisted between both subgroups. iBVP patients seem to recruit brain regions involved in head direction cell processing to a higher extent^38,39^. A better activation of the head direction systems may improve the use of shortcut routes during recombining novel routes (Supplementary Fig. S3). The projection of vestibular pathways to the head direction cell circuit is well known^52,61^. Vestibular signals are transferred from the medial vestibular nucleus (angular head velocity cells) to the dorsal tegmental nucleus, ADN, and from there to the entorhinal cortex and RSC^20,46,47^. In vertebrates, signal input from the SCCs is needed to generate a tuned head direction cell code, whereas otolith input supports the stability of this signal^62,63^. According to the important role of the horizontal SCC for head direction cell tuning, the ADN and RSC showed a navigation-induced activation pattern, which was significantly correlated with both the vHIT gain (high-frequency spectrum of horizontal SCC function) and the caloric response (low-frequency spectrum of horizontal SCC function) (Supplementary Fig. S2). The differential contribution of otolith dysfunction to head direction and place cell circuits could not be further substantiated given that the majority of patients had pathological or absent o/cVEMP responses. In contrast, the observed brain activations in cBVP patients reflect the network for stimulus–response (spatial) learning^28,29^. This seems reasonable, since a complete lack of vestibular information affects the head direction cell code in ADN and RSC, as well as the place cell and grid cell codes within the hippocampus and entorhinal cortex^10–13,64^. A purely age-related effect in iBVP patients is very unlikely, since previous studies showed increased employment of stimulus–response like strategies for absolving spatial tasks with greater age^35,65,66^. In summary, the current study reports additional indirect evidence for the affection of the place cell and head direction cell networks in patients with BVP.

### Strengths and limitations

A major strength of the current study is that it investigated spatial navigation in BVP patients in real space and under naturalistic multisensory feedback rather than within a VR paradigm. Besides the known hippocampal dysfunction in BVP during navigation, it was possible to delineate the role of SCC hypofunction for angular head velocity circuits (in the pontine brainstem) and head direction cell circuits (in the ADN and RSC). As a limitation, [^18^F]-FDG-PET only captures changes of regional glucose metabolism, but does not allow conclusions about the underlying mechanisms (e.g., cell types involved) to be drawn. Navigation-induced activations on retraced familiar routes cannot be disentangled from activations on recombined novel routes due to the integrative nature of [^18^F]-FDG neuronal accumulation. The different layouts of the real-space environment for navigation and the hallway for stereotyped locomotion may confound the interpretation of imaging results. In analysis of eye movements, small saccades below 5 deg might be overlooked by the applied velocity threshold (I-VT) method. However, under the dynamic conditions of our real-space navigation task (with constant small head and eye movements during locomotion) the I-VT approach with a threshold below 5 deg seemed to be best suited to detect saccades while minimizing movement-induced noise. Furthermore, some contribution of hearing loss (present in 6/14 patients) to spatial disorientation cannot be excluded. These patients were, however, well adapted to deafness and received written and drawn instructions.

## Conclusions

The current study expands our knowledge of the contribution of the vestibular system to higher cognitive functions. Recombining novel routes were explicitly affected in BVP, which was paralleled by a disintegration of hippocampal (place cell) and brainstem-thalamic (angular head velocity/head direction cell) networks. Future research is warranted to further clarify the long-term functional consequences of higher vestibular network dysfunction for cognitive domains.

## Methods

### Subjects

Fourteen patients with BVP (according to the diagnostic criteria of the Bárány Society)^67^ and 14 age-matched healthy controls (HC) with normal neurological status and comparable educational levels were included in the study. All subjects underwent a comprehensive neuro-otological examination, including horizontal video head-impulse test (vHIT), caloric cold (32°) and hot (42°) water testing of the horizontal semicircular canals (SCC), ocular and cervical vestibular-evoked myogenic potentials (o/cVEMP), and posturography. Based on these tests, BVP patients were further divided into two subgroups: 6 with complete BVP (cBVP) (no vestibular response from otoliths and SCCs) and 8 with incomplete BVP (iBVP) (with residual vestibular response). Additional hearing loss was documented in the 6 patients with complete BVP (cBVP) due to previous bilateral vestibular neurectomy to treat vestibular schwannomas. Concomitant visual loss (visual acuity < 0.5), or clinically relevant polyneuropathy (vibration sense at the medial malleolus < 5/8) was excluded by clinical neurological examination. Ischemic lesions, microvascular changes (Fazekas > 1), brain atrophy, or other structural brain pathologies were ruled out by structural magnetic resonance imaging (MRI with T2, FLAIR, DWI). Relevant cognitive deficits that might interfere with navigation performance were excluded by Montreal Cognitive Assessment (MOCA).

### Standard protocol approvals and patient consent

Subjects gave their informed, written consent to participate in the study, and for publication of identifying image in an online open-access publication. The protocol was approved by the local Ethics Committee (Ludwig-Maximilians-Universität München) in accordance with the Declaration of Helsinki and the German Federal Office for Radiation Protection.

### Spatial orientation paradigm

All participants performed a well-established navigation paradigm in a complex and unfamiliar spatial environment to test their spatial orientation performance (for details see^21^):

In the exploration phase, the examiner showed the participant the exact location of five different items (pictures of a ball/mushroom/flower/train/house, placed within an outpatient clinic) in a defined sequence. The investigator-guided exploration walk took exactly 10 min for all participants. This was controlled by a stopwatch, which was carried with the investigator. During the exploration phase, the participant followed the investigator to the items in a defined order and along defined routes (start position → ball → mushroom → flower → train → house, and opposite sequence: house → train → flower → mushroom → ball → start position) (Fig. 1). Some items were placed in niches (e.g., ball, flower, house), while others (mushroom, train) were behind doors. The participants had to enter the doors in order to see those items. All items were placed at a height of 1.7 m. The investigator chose a gait velocity of about 1.0 m/s, which allowed controls and patients to reach the items without any discomfort. At the respective target items, a stop of 30 s was included to allow visual exploration. The subjects had to walk a distance of about 330 m to approach all items and return back to the starting point. The participant was instructed to explore the environment intensely, in preparation of the following self-reliant navigation phase.

For the subsequent navigation phase (duration: 10 min), the participants were asked to navigate to the target items by a fully self-determined strategy. They were not motivated to go as fast as possible or as optimal as possible. Subjects were instructed verbally (e.g., “please, go to the ball”) and visually (picture of the ball) to approach the next target item, once they reached the previous one. When a participant did not find the requested target item within a time limit (of 2-times mean duration of the respective route in a previous control sample), the following target item was requested to be approached from the current position. There was no return to the starting position. This strategy was followed consistently in all subjects.

In the first part of the navigation paradigm, the requested sequence of target items was identical to the exploration phase, which means retracing routes familiar from the exploration phase (referred to as retraced familiar routes). In the second part of the navigation paradigm, the target items were requested in a pseudo-randomized order, which required recombining of familiar routes to novel routes (referred to as recombined novel routes). The navigation task was designed to allow use of a possible shortcut route for the recombining of novel routes, which was not actively shown either during the exploration or during the navigation phase. Altogether, the whole real-space navigation task consisted of 15 routes (Fig. 1)^21^. The navigation task included five crossroads. The area of the crossroad was defined as two meters from the crossing point. Distinct spatial clues were eliminated by covering direction signs during the exploration and navigation phase. Each participating subject had to recall the five target items after the exploration and navigation phases to exclude a bias on spatial navigation performance due to (verbal) memory dysfunction. In a previous study, a duration of 10 min was appropriate for healthy controls with a mean gait velocity of 1.1 m/s to complete the 15-route paradigm successfully^21^. If a participant performed outstandingly, and ended the paradigm before the time limit of 10 min, additional target items were presented, which required planning of recombined novel routes (i.e., → train → mushroom → house etc.) (Supplementary Table S2).

The primary outcome parameter was the error rate in both groups (BVP versus HC). An error was registered, when a participant did not find the requested target item within a time limit (2-times mean duration of the respective route in previous control sample)^21^, passed by or ignored the target item. This definition was chosen to avoid a random-like search strategy. If a subject used a non-optimal route but reached the requested item within the above-mentioned time limit, this was accepted. Error rates were normalized to a total of 15 routes in our task (normalized error rate = (15 – correctly found items)/15 × 100%). Some participants with very good navigation performance successfully completed more than 15 routes in 10 min, which resulted in negative normalized error rates (e.g., for 16 correctly approached target items: (15–16)/15 × 100% equals a normalized error rate of − 6.7%). Error rates were further separated for retraced familiar and recombined novel routes and normalized to the number of target items expected to be found in 10 min (retraced familiar routes: n = 5, recombined novel routes: n = 10). The percentage use of the shortcut route was defined by the number of actually used shortcuts/number of all possible shortcuts. The parameter travelled distance/optimal path (%) was added as an indirect measure for the use of inefficient routes (Table 1).

### Recording of navigational path and visual exploration behaviour

All participants wore a gaze-in-space measuring device throughout the experiment to document their visual exploration. This consisted of a mobile infrared video-eye-tracking system with goggles, a head-fixed camera, and an inertial measurement unit with a triaxial accelerometer, gyroscope, and magnetometer to record head movements (for details see^68^). The sampling rate for eye tracking was 220 Hz. A 5th order 21 sample Savitzky-Golay (SG) FIR smoothing filter was applied to preserve high-frequency detail in the signal, while maintaining both temporal and spatial information about local maxima and minima^69^.

Analysis of saccades and fixations was carried out using MATLAB 2012a (Mathworks, Natick, MA, USA) software based on the established algorithm^70^. Raw eye movements in the x and y-axes were converted into degrees in the respective axis and displayed as heat maps. The overall distance traversed by gaze was determined as:

$${\text{Distance}} = \sqrt {\left( {x_{t1} - x_{t2} } \right)^{2} + \left( {y_{t1} - y_{t2} } \right)^{2} }$$

where t_1_ and t_2_ refer to adjacent time points. Velocity was computed as Distance/Time. Acceleration was computed as (Velocity_t1_ − Velocity_t2_)/Time. Saccades were automatically identified by the velocity threshold method (I-VT)^71^ applying a velocity threshold of 240 deg/s and an acceleration threshold above 3000 deg/s^2^. The total frequency of saccades (Hz) was computed. I-VT was applied previously in a similar task setting to quantify saccades under dynamic conditions in older healthy controls and patients with Parkinson's disease^70^. Analysis in our experiment revealed that average saccade rates lay well in the zone of the clinically acceptable 3–5 Hz, suggesting fairly robust performance for saccade identification under dynamic conditions such as walking or navigation. The dynamic task condition of our real-space navigation paradigm with complete freedom for body- and head-rotations, linear accelerations and decelerations, resulted in movement-related small eye movements and VOR-related saccades throughout the task. This background noise did not allow for valid recording of small saccades in the frequency range below 3 Hz, in contrast to static experiments that allow a better control for background signals and noise.

As a fixation we defined, when the gaze was directed towards a certain point or object with a duration of more than 100 ms at a velocity and acceleration cut-off of less than 240 deg/s and 3000 deg/s^2^_,_ respectively. The total frequency of fixations (Hz) was computed quantitatively throughout the whole navigation task. We analyzed overall saccades and fixations for the total task and separately for the retraced familiar and recombined novel routes in a group-wise manner. All saccades and fixations were annotated manually post-hoc to viewed objects by an experienced investigator. In total, 447 viewed separate objects were identified and classified in four major object categories based on their assumed relevance to guide spatial orientation:

- Category 1: non-specific saccades and fixations (e.g., ground floor, bare wall, ceiling)
- Category 2: task-inherent saccades and fixations (e.g., investigator, instructions, target items)
- Category 3: fixations reflecting non-specific general search behaviour (e.g., doors, views into rooms/corners/niches)
- Category 4: object fixations suitable as landmarks for (re)orientation, reflecting specific search behaviour (e.g., distinct fixed pictures, objects and furniture)

By this diligent post-hoc coding of all the object fixations during the exploration and navigation task in each single subject, we were able to depict the average fixation behaviour for both groups (BVP and HC). Therefore, we specifically focused on the object fixations to potential landmarks (category 4, n = 40 objects included). From these objects, we extracted the ones that were fixated at least twice and calculated the mean rate of fixations to single objects on a group level (BVP and HC). The 25 most frequently fixated objects per group were plotted on a ground map of the navigation space as circles (diameter proportionate to the average number of views to the respective object), to depict the spatial distribution of potential landmarks in each group (see Fig. 3a,b). We then analyzed the overlap of object fixations within groups (i.e., BVP-group: exploration versus navigation, HC-group: exploration versus navigation) and between groups (i.e., exploration: BVP- versus HC-group, navigation: BVP- versus HC-group) (see Fig. 3c), to illustrate the selection strategy and recall of landmarks for both groups.

The search path during the navigation task was mapped by accumulating time at a specific place and was analyzed quantitatively for mean gait speed, use of shortcuts, and time spent at crossroads. Gait velocity was calculated in each subject based on the post-hoc coding of the subject position in time. By means of the magnetometer, horizontal head movement velocity (deg/s) was depicted separately throughout the whole task and further differentiated for all the retraced familiar and recombined novel routes.

### [^18^F]-FDG PET imaging of navigation- and locomotion-induced brain activations

To investigate the brain activation pattern during navigation, BVP patients and HC were examined by [^18^F]-FDG-PET following a previously established protocol (for details, see^72,73^): [^18^F]-FDG was injected at the start of the 10-min navigation phase. Afterwards subjects rested in a supine position for 20 min and image acquisition started 30 min after tracer administration (Fig. 1c). Each subject was scanned while in a fasting state > 6 h (checked by means of blood glucose concentration). This paradigm was chosen because the cerebral glucose utilisation is weighted to the 10 min following [^18^F]-FDG injection and is integrative due to intracellular trapping of the tracer^74^. It therefore allows an estimation of neuronal activation specific to the task performed in a time period of 10 min immediately after [^18^F]-FDG injection. A second PET scan was acquired 4 weeks later during hallway locomotion (control condition in a different spatial layout without spatial orientation). Image acquisition was again started 30 min post-injection on an ECAT EXACT HR^+^ PET scanner (Siemens/CTI, Knoxville, TN, USA). The scanner acquires 63 contiguous transaxial planes, simultaneously covering 15.5 cm of an axial field of view. The transaxial and axial resolutions (full width at half maximum) of the PET system were 4.6 mm and 4.0 mm, respectively, at the centre and 4.8 mm and 5.4 mm, respectively, at a radial offset of 10 cm. The patient’s head was secured to a foam cushion and adequately positioned in the gantry. The emission recording consisted of three frames (10 min per frame, 3-D acquisition) covering the period from 30 to 60 min post injection, after which a transmission scan was obtained using a rotating [^68^Ge] point-source. For further evaluation, the frames were added to a single frame (30 min acquisition). Images were reconstructed as 128 × 128 matrices of 2 × 2 mm voxels by filtered back-projection using a Hann filter with a cut-off frequency of 0.5 Nyquist and corrected for random, dead time, scatter, and attenuation. The reconstructed [^18^F]-FDG images were transformed to NIfTI format for further processing.

### Analysis of [^18^F]-FDG PET data acquired during navigation and locomotion

Data processing and statistical analysis were performed using SPM8 software (Wellcome Department of Cognitive Neurology, London) implemented in MATLAB 2012a following an established protocol^21,72^. All the reconstructed [^18^F]-FDG-PET images were linearly co-registered to the corresponding MRI using automated SPM8 algorithms. Anatomical brain MRIs were spatially normalised into the MNI standard template (McGill University, Montreal QC, Canada) using an affine transformation (12 parameters for rigid transformations), whose parameters were applied to the co-registered [^18^F]-FDG-PET images. Then the spatially normalised images were blurred with a Gaussian filter (FWHM 12 mm) to account for regional inter-subject variability. All scans were analyzed after normalisation to the white matter^72,73^. The normalisation prior to voxel-based statistics was done with an anatomical mask (centrum semiovale) in MNI space, to remove the effects of different overall counts. Images of the spatial orientation paradigms were compared voxel-wise with those of the control condition (paired t-test) and between groups (unpaired t-test). Correlation analyses of regional cerebral glucose metabolism (rCGM) were done with vestibular function tests (mean vHIT gain, mean caloric response), normalized error rates, % use of the shortcut route, duration at crossroads, and object-specific fixation frequency on recombined novel routes. rCGM increases and decreases were considered significant for a p < 0.005, similar to previous studies^21^.

### Statistical analysis

Behavioural and navigational measurements of the three groups (cBVP, iBVP, HC) were analyzed using SPSS 24 (IBM, Armonk New York). Independent sample t-tests were used to compare the demographic data, navigation performance, visual search parameters, search path (i.e., duration at crossroads, use of shortcut route), head movements, and gait speed between BVP patients and HC. Kolmogorov–Smirnov was applied to guarantee the normal distribution of all the parameters analyzed by the independent sample t-test. To correct for multiple testing of all the different eye movement (i.e., fixations and saccades) and head movements parameters from Table 1, Bonferroni-correction was applied post hoc. P-values < 0.05 were considered statistically significant. Spearman’s rank correlation was analyzed for normalized error rates during navigation against the degree of vestibular impairment (quantified by mean vHIT gain, mean caloric response) and considered significant for Rho > 0.5 and p < 0.05.

## Supplementary Information

Supplementary Information.

## Data Availability

Data reported in this article will be shared with any appropriately qualified investigator on request after pseudonymization.

## Abbreviations

ADN: Anterior dorsal nucleus of the thalamus
cBVP: Complete bilateral vestibulopathy
iBVP: Incomplete bilateral vestibulopathy
[^18^F]-FDG: [^18^F]-fluorodeoxyglucose
HC: Healthy controls
MNI: Montreal neurological institute
MOCA: Montreal cognitive assessment
MRI: Magnetic resonance imaging
MWMT: Morris water maze task
PET: Positron emission tomography
PPA: Parahippocampal place area
PPC: Posterior parietal cortex
rCGM: Regional cerebral glucose metabolism
RSC: Retrosplenial cortex
SCC: Semicircular canal
SPM: Statistical parametric mapping
SPSS: Statistical package for the social sciences
VEMP: Vestibular evoked myogenic potential
VR: Virtual reality
vHIT: Video-head-impulse test
