# Supplementary material for: Bilateral vestibulopathy causes selective deficits in recombining novel routes in real space
Source: Sci Rep. 2021 Jan 29;11:2695. doi: 10.1038/s41598-021-82427-6 (PMC7846808; doi:10.1038/s41598-021-82427-6)
Supplement: Supplementary file 1 — Supplementary Information. [file 41598_2021_82427_MOESM1_ESM.docx]

**Bilateral vestibulopathy causes selective deficits in recombining novel routes in real space**

Florian Schöberl^1,2^, MD, Cauchy Pradhan^2^, MD, PhD, Maximilian Grosch^2^,

Matthias Brendel^3^, MD, Florian Jostes^1^, Katrin Obermaier^1^, Chantal Sowa^1^, Klaus Jahn^2,4^, MD, Peter Bartenstein^3^, MD, Thomas Brandt^2,5^, MD, Marianne Dieterich^1,2,6^ , MD, Andreas Zwergal^1,2,*^, MD

^1^ Department of Neurology, University Hospital, LMU Munich, Germany

^2^ German Center for Vertigo and Balance Disorders, DSGZ, LMU Munich, Germany

^3^ Department of Nuclear Medicine, LMU Munich, Germany

^4^ Neurological Hospital, Schön Klinik Bad Aibling, Germany

^5^ Clinical Neurosciences, LMU Munich, Germany

^6^ Munich Cluster of Systems Neurology, SyNergy, Munich, Germany

*** Corresponding author**: Andreas Zwergal, MD, Department of Neurology and German Center for Vertigo and Balance Disorders, DSGZ, Ludwig-Maximilians-University Munich,

Marchioninistrasse 15, D-81377 Munich, Germany

Phone: +49 89 4400 72571, Fax: +49 89 4400 75584

E-Mail: [andreas.zwergal@med.uni-muenchen.de](mailto:andreas.zwergal@med.uni-muenchen.de)

**SUPPLEMENTARY INFORMATION**

**Supplementary Table S1**

| **ID** | **Age** | **Gender** | **Etiology** | **Mean bilateral vHIT gain** | **Mean bilateral caloric response (deg/sec)** | **cVEMP** | **oVEMP** |
| --- | --- | --- | --- | --- | --- | --- | --- |
| 1 | 44 | m | NF-2 | 0 | 0 | Abnormal | Abnormal |
| 2 | 41 | f | NF-2 | 0 | 0 | Abnormal | Abnormal |
| 3 | 45 | f | NF-2 | 0 | 0 | Abnormal | Abnormal |
| 4 | 47 | m | NF-2 | 0 | 0 | Abnormal | Abnormal |
| 5 | 40 | f | NF-2 | 0 | 0 | Abnormal | Abnormal |
| 6 | 62 | f | NF-2 | 0 | 0 | Abnormal | Abnormal |
| 7 | 45 | m | Toxic | 0.5 | 2.0 | Normal | Abnormal |
| 8 | 58 | m | Toxic | 0.4 | 1.3 | Abnormal | Abnormal |
| 9 | 42 | f | Toxic | 0.4 | 2.0 | Abnormal | Abnormal |
| 10 | 69 | m | Idiopathic | 0.2 | 0.7 | Abnormal | Abnormal |
| 11 | 76 | m | Idiopathic | 0.6 | 4.0 | Normal | Normal |
| 12 | 68 | f | Idiopathic | 0.5 | 3.0 | Abnormal | Normal |
| 13 | 58 | m | Idiopathic | 0.4 | 2.0 | Abnormal | Abnormal |
| 14 | 72 | f | Idiopathic | 0.3 | 2.0 | Abnormal | Normal |

**Supplementary Table S1:** Patient characteristics and neuro-otological test parameters. Six patients had a complete BVP following bilateral vestibular neurectomy (due to vestibular schwanommas), 8 patients an incomplete BVP (toxic, idiopathic etiologies). BVP: bilateral vestibulopathy, NF-2: neurofibromatosis type 2, vHIT: video head impulse test, cVEMP: cervical vestibular evoked myogenic potential, oVEMP: ocular vestibular evoked myogenic potential.

**Supplementary Table S2**

|  | **Navigation target items** | **Routes** | **Shortcut route appropriate** | **Probability of success by random search** |
| --- | --- | --- | --- | --- |
| 1 | Ball | Retraced / familiar | No | 0.5 |
| 2 | Mushroom | Retraced / familiar | No | 0.25 |
| 3 | Flower | Retraced /familiar | No | 0.13 |
| 4 | Train | Retraced /familiar | No | 0.13 |
| 5 | House | Retraced /familiar | No | 0.5 |
| 6 | Mushroom | Recombined /novel | Yes | 0.0625 – 0.13 |
| 7 | Ball | Recombined /novel | No | 0.25 |
| 8 | Train | Recombined /novel | Yes | 0.0625 – 0.13 |
| 9 | Flower | Recombined /novel | No | 0.13 |
| 10 | House | Recombined /novel | No | 0.13 |
| 11 | Mushroom | Recombined /novel | Yes | 0.0625 – 0.13 |
| 12 | Train | Recombined /novel | Yes | 0.0625 – 0.13 |
| 13 | Ball | Recombined /novel | Yes | 0.0625 – 0.13 |
| 14 | House | Recombined /novel | Yes | 0.0625 – 0.13 |
| 15 | Ball | Recombined /novel | Yes | 0.0625 – 0.13 |
| *16* | *Train* | *Recombined / novel* | *Yes* | *0.0625 – 0.13* |
| *17* | *Mushroom* | *Recombined / novel* | *Yes* | *0.0625 – 0.13* |
| *18* | *House* | *Recombined / novel* | *Yes* | *0.0625 – 0.25* |

**Supplementary Table S2:** Probability of successful route finding by random search.

**Supplementary Table S3**

| **Parameter** | **HC** | **BVP** | **Independent t-test (t,p)** |
| --- | --- | --- | --- |
| Total saccades during standing | 318.2 ±210.0 | 413.7 ± 16.7 | 1.3, 0.80 |
| Familiar routes: saccades during standing | 93.4 ±84.2 | 145.0±61.6 | 1.5, 0.80 |
| Novel routes: saccades during standing | 224.7±137.2 | 268.7±51.0 | 0.9, 0.80 |
| Total fixations during standing | 176.7±119.2 | 293.7±26.8 | 2.7, 0.17 |
| Familiar routes: fixations during standing | 51.9±47.9 | 102.3±36.0 | 2.5, 0.22 |
| Novel routes: fixations during standing | 124.8±137.2 | 191.3±43.0 | 2.3, 0.29 |
| Overall horizontal head movement velocity during standing (deg/sec) | 24.9 ± 7.3 | 19.7 ± 3.1 | 1.9, 0.55 |
| Familiar routes: horizontal head movement velocity during standing (deg/sec) | 30.3 ± 4.4 | 23.7 ± 1.6 | 1.6, 0.80 |
| Novel routes: horizontal head movement velocity during standing (deg/sec) | 21.9 ± 6.4 | 16.3 ± 2.0 | 2.4, 0.28 |
| Overall horizontal head movement velocity during walking (deg/sec) | 12.4 ± 2.4 | 10.4 ± 3.1 | 1.6, 0.80 |
| Familiar routes: horizontal head movement velocity during walking (deg/sec) | 13.7 ± 4.1 | 12.8 ± 5.9 | 4.4, 0.80 |
| Novel routes: horizontal head movement velocity during walking (deg/sec) | 12.0 ± 3.0 | 9.3 ± 1.6 | 2.3, 0.29 |

**Supplementary Table S3:** Comparison of number of saccades, fixations, and head movement velocity during standing and walking periods in HC and BVP. Independent t-test between groups with post-hoc correction showed no significant difference for any parameter between groups. BVP: bilateral vestibulopathy, HC: healthy controls.

**Supplementary Figure S1**

**
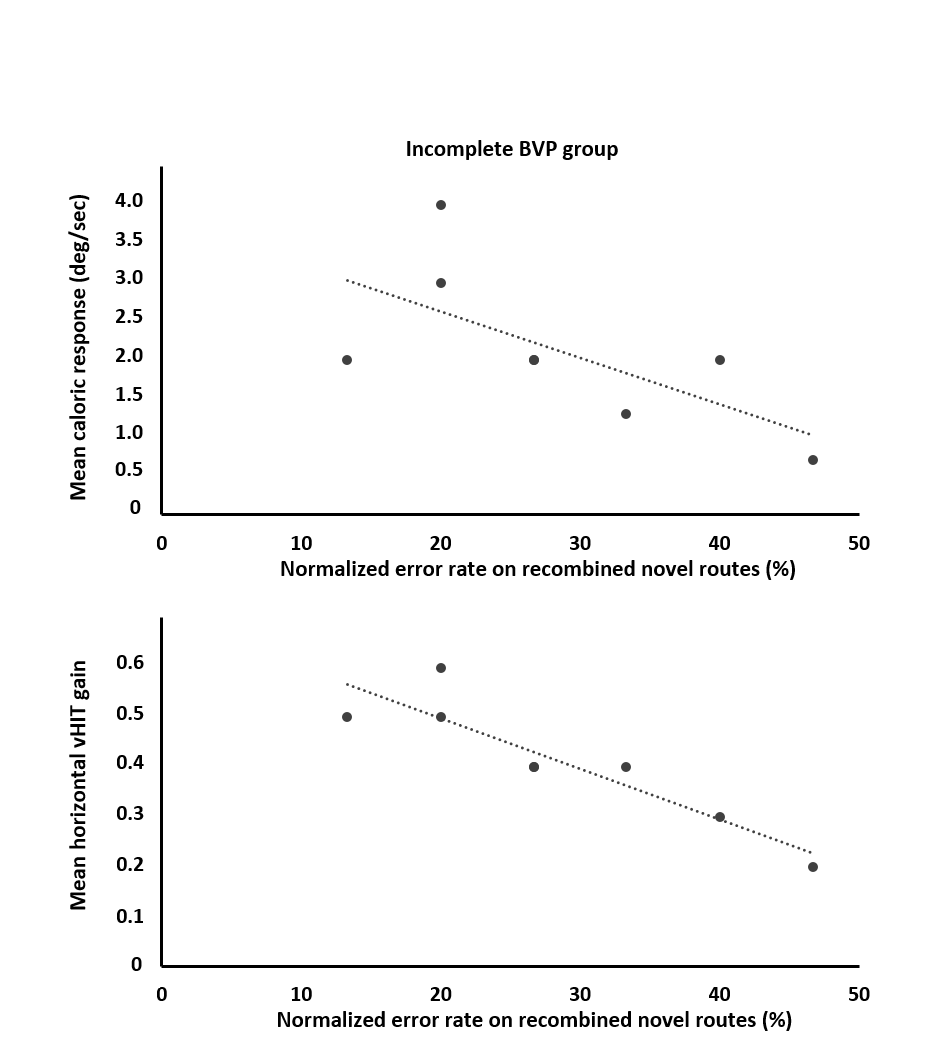
**

**Supplementary Figure S1:** Negative correlation of mean caloric response (Rho = -0.66) and horizontal vHIT gain (Rho = -0.91) with normalized error rate on recombined novel routes in the group of patients with incomplete BVP (n = 8). Please notice that data points of two patients were identical and therefore superposed (mean caloric response of 2.0 deg/sec, normalized error rate of 26.7%; mean horizontal vHIT gain of 0.4, normalized error rate of 26.7%). BVP: bilateral vestibulopathy, vHIT: video head impulse test.

**Supplementary Figure S2**


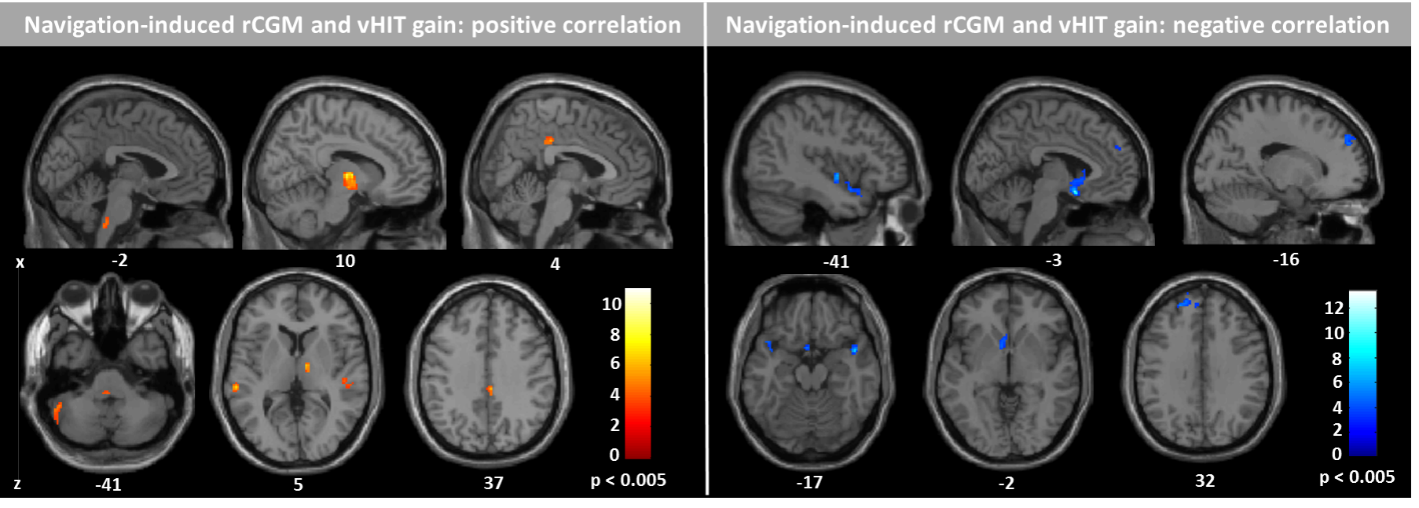


**Supplementary Figure S2:** Correlation analysis of regional cerebral glucose metabolism (rCGM) during navigation and degree of vestibulo-ocular reflex deficits in BVP patients. Navigation-induced rCGM increased with mean horizontal vHIT gain in the pontine brainstem tegmentum, left flocculus, right anterior thalamus, retrosplenial cortex, and bilateral superior temporal gyrus (left side). rCGM during navigation decreased with vHIT gain in the orbitofrontal cortex, left prefrontal cortex, and left caudate nucleus (right side). Significance level p < 0.005; levels of sections in x- and z-direction are given by MNI coordinates. rCGM: regional cerebral glucose metabolism, vHIT: video head impulse test.

**Supplementary Figure S3**


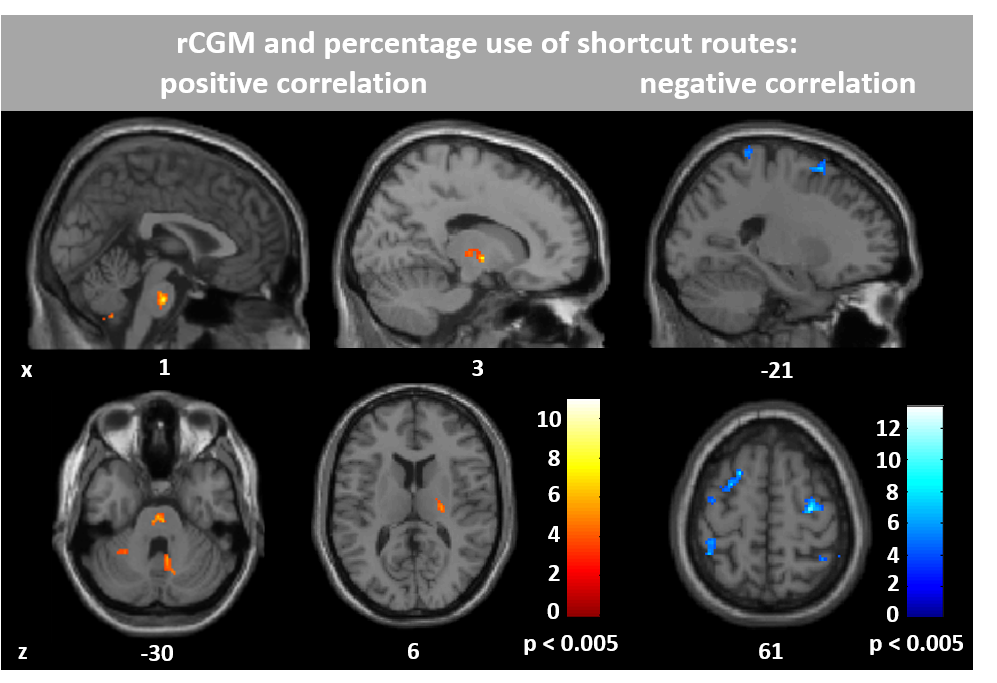


**Supplementary Figure S3:** Correlation analysis of regional cerebral glucose metabolism (rCGM) during navigation and percentage use of shortcut routes in BVP patients. Navigation-induced rCGM increased with more frequent use of shortcut routes in the pontine brainstem tegmentum, left flocculus, right vermis, and right anterior and lateral thalamus (left side). rCGM during navigation decreased with more frequent use of shortcut routes in the bilateral prefrontal cortex (right side). Significance level p < 0.005; levels of sections in x- and z-direction are given by MNI coordinates. rCGM: regional cerebral glucose metabolism.
